# IGF-1R Inhibition Suppresses Cell Proliferation and Increases Radiosensitivity in Nasopharyngeal Carcinoma Cells

**DOI:** 10.1155/2019/5497467

**Published:** 2019-07-31

**Authors:** Zhe Wang, Guangyan Liu, Jiwei Mao, Min Xie, Ming Zhao, Xuefen Guo, Shanshan Liang, Heming Li, Xuefeng Li, Ruoyu Wang

**Affiliations:** ^1^Department of Medical Oncology, Affiliated Zhongshan Hospital of Dalian University, Dalian 116001, China; ^2^The Key Laboratory of Biomarker High Throughput Screening and Target Translation of Breast and Gastrointestinal Tumor, Dalian University, Dalian 116001, China; ^3^College of Basic Medical Sciences, Shenyang Medical College, Shenyang 110034, China; ^4^The Sixth Affiliated Hospital of Guangzhou Medical University, Qingyuan People's Hospital, Sino-French Hoffmann Institute, School of Basic Medical Sciences, Guangzhou Medical University, Guangzhou 511436, China; ^5^Shenzhen Luohu People's Hospital, The Third Affiliated Hospital of Shenzhen University, Shenzhen 518001, China; ^6^Key Laboratory of Regenerative Biology, Guangdong Provincial Key Laboratory of Stem Cell and Regenerative Medicine, South China Institute for Stem Cell Biology and Regenerative Medicine, Guangzhou Institutes of Biomedicine and Health, Chinese Academy of Sciences, Guangzhou 510530, China

## Abstract

Although ionizing radiation (IR) has provided considerable improvements in nasopharyngeal carcinoma (NPC) treatment, radioresistance is still a major threat for some subsets of patients. The insulin-like growth factor-1 receptor (IGF-1R) signaling pathway is tightly regulated and plays critical roles in mediating cell proliferation, growth, and survival. Thus, IGF-1R may be a potential therapeutic target for patients with different malignancies. However, its mechanism in NPC is not fully investigated. Linsitinib is an oral small molecule and is a tyrosine kinase inhibitor (TKI) of IGF-1R, which has been known for antitumor effects used widely. Here, we evaluated the proliferation and radiosensitivity of NPC cell lines (CNE-2 and SUNE-1) after linsitinib treatment. We found that linsitinib suppresses IGF-1-induced cell proliferation through inhibiting Akt and ERK phosphorylation. Moreover, linsitinib further boosted IR-induced DNA damage, G2-M cell cycle delay, and apoptosis in NPC cells. Finally, linsitinib reversed radioresistant NPC cells by decreasing the phosphorylation of IGF-1R. Our data indicated that the combination of linsitinib and IR and targeting IGF-1R by linsitinib could be a promising therapeutic strategy for NPC.

## 1. Introduction

Nasopharyngeal carcinoma (NPC) is a malignancy that arises in the epithelial cells of the nasopharynx [1]. Despite its low incidence with less than 1 per 100,000 in Europe and USA, NPC is of high occurrence in southeast Asia, particularly in southern China with a rather high incidence: 60 per 100,000 and mortality of 34 per 100,000 in 2015 [2, 3]. Accordingly, dietary factors as well as Epstein-Barr virus infection contribute to the development of NPC [4]. Two-dimensional (2D) radiotherapy, three-dimensional (3D) radiotherapy, and intensity-modulated radiotherapy (IMRT) have shown optimistic outcomes for NPC patient, with five-year overall survival (OS) 71%, 73%, and 80%, respectively [5]. Even with treatment, there are still 20-30% NPC patient suffering from local recurrence and short-term disease out control after IMRT [6]. Thus, radioresistance, recurrence, distant failure, and acute and chronic oral complications caused by ionizing radiation (IR) remain the key challenges [7]. The development of molecular-targeted therapy over the past decades provides a beneficial option for NPC treatment. Some reagents, such as the anti-EGFR antibody, cetuximab, the anti-VEGF antibody, and bevacizumab, have been subjected to clinical usage against NPC [8, 9]. However, a pertinent concern of bevacizumab is the increased risk of bleeding [10]. High incidence of grade 3-4 mucositis (87%) and grade 3 radiotherapy-related dermatitis (20%) has also been observed in NPC patients treated with cetuximab [11]. Therefore, finding new regimen to provide effective therapeutics is of great need for NPC treatment.

IGF-1R is a ubiquitous growth receptor, which is involved in the regulation of proliferation, apoptosis, differentiation, and malignant transformation of cancer cells [12]. IGF-1R induces autophosphorylation and activation of specific tyrosine kinase residues, initiating signaling cascades such as Ras/Raf/mitogen-activated protein kinases (MAPK) and phosphoinositide 3-kinase (PI3K), which are downstream oncoproteins involved in many cellular activities [13]. IGF-1R has been reported to be associated with an aggressive clinical course and resistance to chemotherapy and targeted agents [14–16]. As a predictive marker, IGF-1R has been demonstrated to be associated with tumor grade and poor survival in a variety of solid tumors in many studies [17–20]. Elevated serum level of IGF-I results in overactivation of mitogenic, antiapoptotic, and promotility signaling cascades and has been implicated in tumorigenesis, including lung cancer, prostate cancer, and breast cancer [21, 22]. Recent studies revealed that blocking IGF-1R pathway, such as small molecule tyrosine kinase inhibitor (TKI, linsitinib) and monoclonal antibodies, can exert attractive effects for the treatment of various types of cancer in clinical trials [23]. However, few studies investigated the efficacy of IGF-1R inhibition in NPC, and the cellular side effects of linsitinib combined with IR have never been tested in NPC cells (NPCs). Besides, the improvement of NPC survival is limited by traditional therapeutics. Thus, IGF-1R inhibition mechanism by linsitinib is worthy to be evaluated and demonstrated in details.

In the present study, we utilized linsitinib to investigate the antiproliferation effects on NPCs. And we demonstrated that linsitinib sensitizes IR-treated NPCs through persistent DNA damage, cell cycle arrest, and apoptosis induction. Finally, we propose that the combination of linsitinib and IR may lead to significant clinical benefits and provide the basis for further development of targeted therapeutics for NPC.

## 2. Materials and Methods

### 2.1. Cell Culture and Reagents

Five human NPC cell lines (CNE-1, CNE-2, SUNE-1, 5-8F, and 6-10B) were kindly provided by Prof. Yunfei Xia (Sun Yat-Sen University Cancer Center, Guangzhou, China). NPC cell lines were maintained in RPMI-1640 supplemented with 10% fetal bovine serum (FBS), 100 units/ml penicillin, 100 mg/ml streptomycin, and 2 mM of glutamine and cultured at 37°C with a humidified 5% CO_2_.

The linsitinib (IGF-1R inhibitor) was obtained from Selleckchem (Houston, TX, USA) and dissolved in DMSO (Sigma-Aldrich) at a concentration of 10 mM. 0.1% DMSO was used to be a control treatment of 10 *μ*M linsitinib. Recombinant human IGF-I was purchased from R&D System (Wiesbaden, Germany).

### 2.2. Cell Proliferation Assay

Cell proliferation assay was performed using the 3-(4,5-dimethylthiazol-2-yl)-2,5-diphenyl tetrazolium (MTT) dye reduction method. In brief, cells were seeded at a density of 2 × 10^3^ cells/well in 96-well plates and incubated for 24 h. The cells were then treated with DMSO as a control and indicated concentration of linsitinib, IGF-I, and the combination of linsitinib and IGF-1. After 72 h incubation, 50 *μ*l of MTT (5 mg/ml; Sigma-Aldrich) was added to the plate and incubated for another 4 h in 37°C. Then, MTT solution was removed and 100 *μ*l DMSO was added to stop the reaction. The absorbance was measured at 570 nm with a microplate reader. Results were presented as the percentage of untreated group. Each experiment was performed in triplicate at least three times, independently.

### 2.3. Immunoblot Analysis and Antibodies

Western blotting was performed as previously described [24]. The primary antibodies used in this study were rabbit anti-IGF-1R (9750s), phospho-IGF-1R (3021s), ERK (9102s), phospho-ERK (9101s) (1 : 1000 each; Cell Signaling Technology, Danvers, MA); AKT (sc-5298) and phospho-AKT (sc-7985-R) (1 : 1000 each; Santa Cruz Biotechnology); and mouse anti-*γ*H2AX (phosphorS139; 1 : 500; Abcam, San Francisco, CA, USA).

### 2.4. Cell Cycle and Apoptosis Assay

Cells were seeded in 6-well plates and treated with linsitinib (0.4 nM) for 1 h before IR (4 Gy), and then, cells were harvested for another 24 h after IR. Cell cycle distribution was analyzed by propidium iodide (PI) staining and flow cytometry. Briefly, after 24 h of IR, cells were collected, gently washed with cold PBS containing 2% FBS, fixed in 70% cold ethanol, and stored at -20°C overnight. Cells were then pelleted, washed, and stained with PI/ribonuclease staining reagents (BD Biosciences) for 15 min at room temperature. Apoptosis was measured with annexin V-FITC apoptosis detection kit (Vazyme, Najing, China) according to the manufacturer's protocols. Analysis was performed on the FACSCalibur using CellQuest software (BD FACS Aria). All experiments were performed at least three times.

### 2.5. Clonogenic Survival Assays

CNE-2 and SUNE-1 cells were seeded in 6-well plates with indicated density. NPC cells were pretreated with DMSO as control or with linsitinib and/or IGF-I for 1 h and then treated with increasing doses of IR (0, 2, 4, 6, and 8 Gy). The cells were washed 24 h after irradiation. After 10-14 days, the cells were fixed with 4% paraformaldehyde and stained with 0.2% crystal violet. The colonies of at least 50 normal appearing cells were scored as survivors. The data was analyzed using the single-hit multitarget model. Survival fractions (SF) were fitted to the following single-hit multitarget formula: SF = 1 − (1 − *e*^−*D*/D0^)^*N*^, by using GraphPad Prism 5.0 software (GraphPad, La Jolla, CA).

### 2.6. Establishment of Radioresistant Cell Line

CNE-2 cells were plated in 75 cm^2^ culture flask and irradiated with 4 Gy X-ray (Elekta Synergy platform, Sweden) at a dose rate of 400 cGy/min at room temperature when the cell grew to ~80% confluence. The fresh culture medium was changed after irradiation. CNE-2 cells were treated with 4 Gy again when it reached ~80% confluence. These procedures were repeated up to the total dose of 80 Gy for 4 months.

### 2.7. Statistical Analysis

All data were presented as means ± SD from triplicate. The experiments were repeated at least three times independently. The significant difference between different groups was measured by the two-tailed unpaired Student's *t*-test or one-way ANOVA. For all statistical analysis, *P* < 0.05 was considered as significant.

## 3. Results

### 3.1. IGF-1R Inhibition Suppresses Cell Proliferation and IR Induces Phosphorylation of IGF-1R in NPC Cell Lines

**Figure 1:**
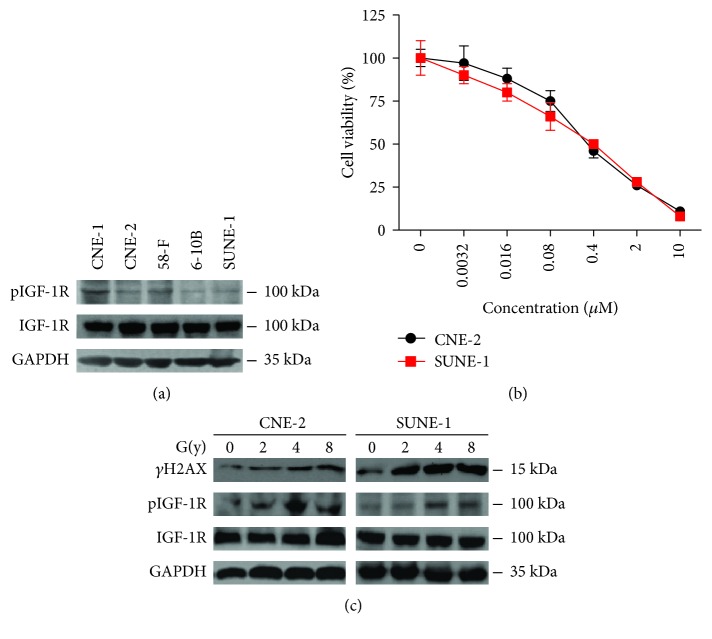
pIGF-1R was activated by ionizing radiation, and linsitinib suppressed cell proliferation in NPC cells. (a) Basal levels of total and phosphorylated IGF-1R in NPC cell lines. (b) Linsitinib suppressed NPC cell proliferation. CNE-2 and SUNE-1 cells were incubated with increasing concentrations of linsitinib, and cell growth was determined after 72 h treatment by MTT assay. (c) CNE-2 and SUNE-1 cells were treated with IR. Cell lysates were harvested at 24 h post irradiation, and the indicated proteins were determined by Western blotting.

We first detected basal levels of the total and phosphorylated IGF-1R (pIGF-1R) in five NPC cell lines. All five cell lines presented different levels of pIGF-1R: CNE-1 and 5-8F were the highest, CNE-2 and SUNE-1 were intermediate, and 6-10B was the lowest (Figure 1(a)). To find the proper working concentration of linsitinib, we performed cell proliferation assays. Rather than other cell lines, both CNE-2 and SUNE-1 cells were sensitive to linsitinib (IC50 = 0.387 *μ*M and 0.381 *μ*M, respectively) (Figure 1(b)). Thereby, we chose 0.4 *μ*M linsitinib in the following experiments. IR could induce DNA breakage and induce DNA damage response. *γ*H2AX is a standard marker to indicate IR application [25]. Consistent with previous results, we found that IR boosted *γ*H2AX expression. Meanwhile, IR significantly increased pIGF-1R in a dose-dependent manner but failed to affect basal IGF-1R (Figure 1(c)). In addition, 4 Gy IR leads to a maximum phosphorylation of IGF-1R in CNE-2 and SUNE-1 cells. These results demonstrated that linsitinib suppressed cell proliferation and IR can over activate IGF-1R phosphorylation, suggesting that pIGF-1R is an operative receptor in NPCs.

### 3.2. IGF-I Enhances Cell Proliferation via Activating IGF-1R

**Figure 2:**
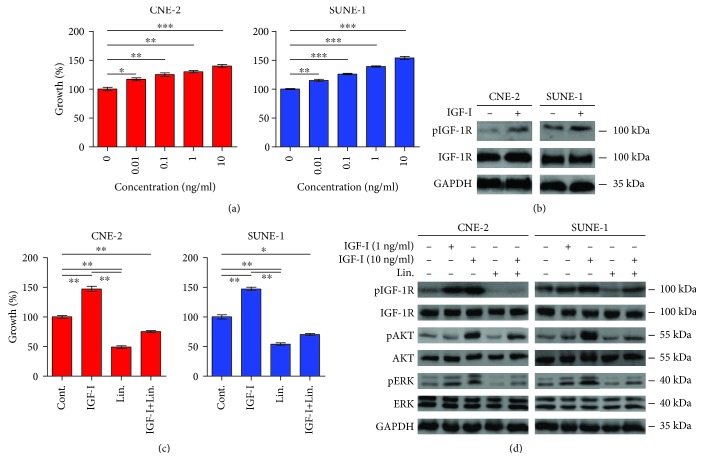
IGF-I promotes cell proliferation by activating pIGF-1R, which can be reversed by linsitinib. (a) Serum-starved CNE-2 and SUNE-1 cells were incubated with increasing concentration of IGF-I, and the cell growth was determined after 72 h treatment by MTT assay. (b) The serum-starved CNE-2 and SUNE-1 cells were cultured overnight and then treated with IGF-1 (10 ng/ml) for 1 h. The cell lysates were harvested and subjected to Western blotting. (c) The serum-starved cells were pretreated with or without linsitinib (Lin., 0.4 *μ*M) for 2 h followed by IGF-I (10 ng/ml) stimulation for 72 h. Then, cell growth was determined by MTT assay. (d) The serum-starved cells were pretreated with or without linsitinib (Lin., 0.4 *μ*M) for 2 h followed by indicated IGF-I stimulation for 1 h, and then, cell lysates were subjected to Western blotting. ^∗^*P* < 0.05; ^∗∗^*P* < 0.01.

Previous studies have reported that the IGF-I/IGF-1R axis plays a role in tumor development and progression. Upregulated IGF-1R or its ligands have been observed in several tumor types [17–20]. Accordingly, we next detected the effect of IGF-I, a IGF-1R ligand, on the proliferation of NPCs. IGF-I significantly increased the proliferation of these cells in a dose-dependent manner (Figure 2(a)). Western blotting confirmed that IGF-I apparently increased the phosphorylation of IGF-1R in NPCs, but not the total IGF-1R (Figure 2(b)). Inhibition of IGF-1R by small molecule inhibitor has been shown to prevent tumor growth in mice xenograft models [26]. Consistent with previous studies, linsitinib completely blocked IGF-I-induced cell proliferation in our experiments (Figure 2(c)). In order to dissect the mechanisms of the underlined signaling pathways that control cell proliferation, we determined the expression of Akt and ERK, which are downstream-regulated proteins in IGF-I/IGF-1R axis. Consistent with previous studies in other cells, we found that IGF-I increased the phosphorylation of IGF-1R in a dose-dependent manner (Figure 2(d)). Meanwhile, IGF-I also induced the phosphorylation of Akt (pAkt) and ERK (pERK) in NPCs, which play crucial roles in activating MAPK and PI3K signaling pathways (Figure 2(d)). Linsitinib slightly decreased the phosphorylation of ERK but not Akt. Notably, linsitinib completely conceal the effect of IGF-I-induced phosphorylation of IGF-1R, ERK, and Akt in NPCs, which might be linked with cell proliferation. These findings suggested that the IGF-I/IGF-1R signaling pathway is critical for cell proliferation in NPCs and linsitinib could effectively block IGF-I/IGF-1R signaling pathway, consequently inhibiting MAPK and PI3K signaling pathways.

### 3.3. Targeting IGF-I/IGF-1R by Linsitinib Increases the Radiosensitivity of NPC Cells

**Figure 3:**
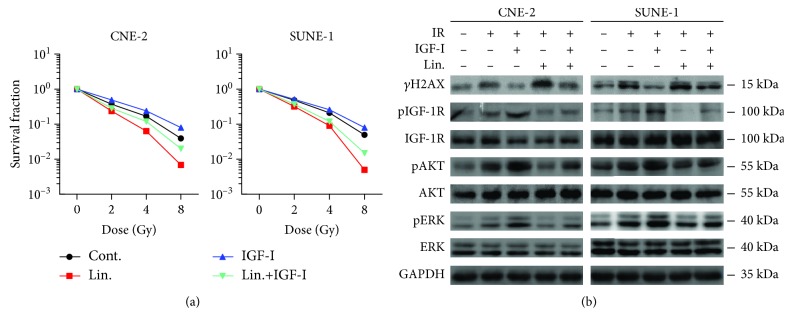
Targeting IGF-I/IGF-1R by linsitinib increases the radiosensitivity and reverses the radioresistance induced by IGF-I in NPC cells. (a) The clonogenic survival assays of CNE-2 and SUNE-1 cells. Cells were treated with linsitinib (Lin., 0.4 *μ*M) and/or IGF-I (10 ng/ml) followed by irradiation in a range of radiation doses. Each data point represents the mean of three experiments ± SD. (b) Response of PI3K/AKT and MAPK/ERK signaling pathways. The serum-starved CNE-2 and SUNE-1 cells were cultured overnight. 1 h prior IR (4 Gy), cells were treated as indicated. The cell lysates were harvested at 1 h post IR and the phosphorylation of indicated proteins was determined by Western blotting. ^∗∗^*P* < 0.01.

Since IGF-1R has been implicated in cell proliferation responses, we hypothesized that the combination of IGF-1R blockade and IR would increase the radiosensitivity of NPC cells. As expected, the combination of IR and linsitinib significantly reduced the survival fraction in both CNE-2 and SUNE-1 cells (Figure 3(a), red dot *vs.* black dot). IGF-I addition increased the IR-treated cell survival fraction in NPCs (Figure 3(a) blue dot *vs.* black dot). Strikingly, the administration of linsitinib reversed the IGF-I-induced radioresistance in NPCs (Figure 3(a) cyan dot *vs.* blue dot). *γ*H2AX is involved in recruiting DNA repair proteins in response to DNA double-strand breaks (DSBs) and has been reported as a biomarker of DNA damage. Moreover, IGF-1R inhibition delayed the resolution of irradiation-induced DSBs in prostate cancer cells [27]. Under our experiment condition, IGF-I addition suppressed IR-induced *γ*H2AX expression, whereas the administration of linsitinib enhanced IR-induced *γ*H2AX expression compared with the cells exposed to IR alone. Moreover, linsitinib abrogated the effect of *γ*H2AX induction upon IGF-I addition (Figure 3(b)).

To investigate the molecular mechanism of how linsitinib increases radiosensitivity in NPCs, we examined the phosphorylation of IGF-1R, Akt, and ERK by Western blotting. The combination of IR and IGF-I increased the phosphorylation of Akt and ERK. However, linsitinib reversed IGF-I and IR induced pAkt and pERK in NPCs (Figure 3(b)). These results clearly demonstrated that linsitinib, as an IGF-1R inhibitor, increased the radiosensitivity and reversed the radioresistance induced by IGF-I in NPCs. And these might be associated with the persistence of DNA damage through Akt or ERK inhibition.

### 3.4. The Combination of IR and pIGF-1R Inhibitor Induces Cell Cycle Arrest and Apoptosis in NPC Cells

**Figure 4:**
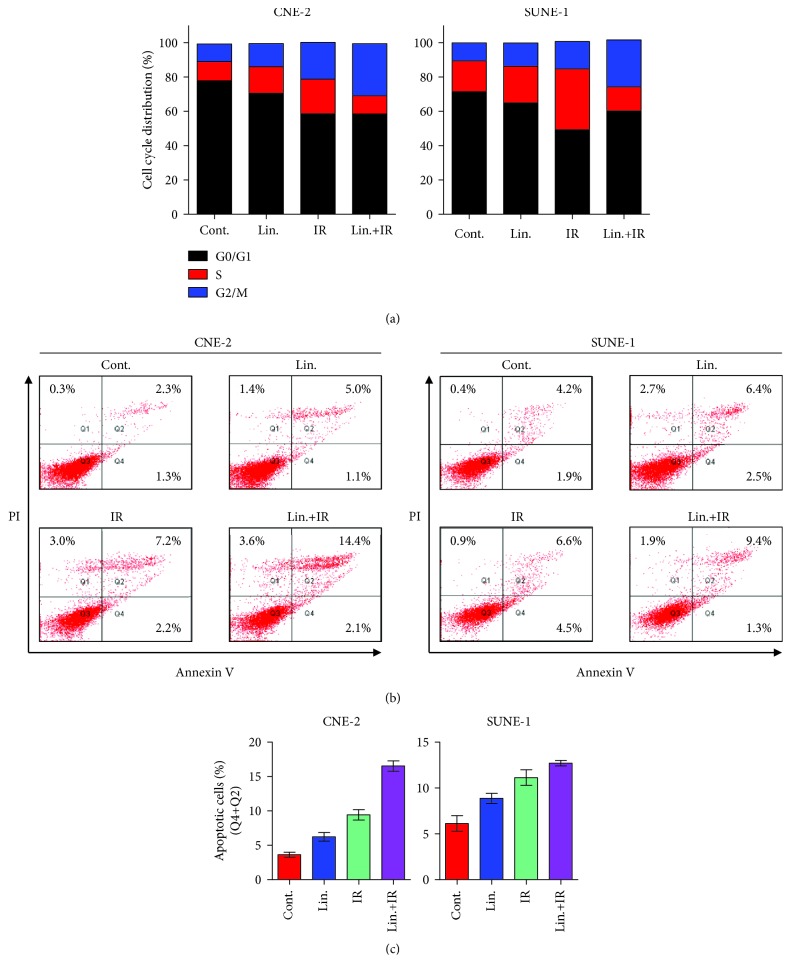
Linsitinib with or without IR induces cell cycle arrest and apoptosis in NPC cells. (a) CNE-2 and SUNE-1 cells were treated with linsitinib (Lin., 0.4 *μ*M) and/or IR (4 Gy) for 24 h. Then, cells were harvested and stained with propidium iodide (PI) staining, and cell cycle was determined by flow cytometry. (b) CNE-2 and SUNE-1 cells were treated as cell apoptosis assay and stained with annexin V-FITC/PI. The quantification of the percentage of apoptotic cells includes early apoptotic (bottom right quarter) and late apoptotic (top right) cells. (c) The statistical graph of (b). Each experiment was repeated at least three times.

To investigate whether linsitinib radiosensitizes NPC cells via redistributing cell cycle, we performed cell cycle assay on NPC cells exposed to IR with or without linsitinib treatment. Linsitinib alone led to an increased S phase percentage, whereas IR alone led to a G2-M arrest. Strikingly, the combination of IR and linsitinib induced a further arrest of G2-M phase, which implicates the potential radiosensitizing capability of linsitinib (Figure 4(a)). In addition, treatment with IR (9.4%) and linsitinib (6.1%) alone could induce apoptosis in CNE-2 cells as compared with normal cells (3.6%). Notably, the combination of linsitinib and IR could induce apoptosis rate up to 16.5%. Similar results were obtained in SUNE-1 cells (Figures 4(b) and 4(c)). Taken together, our results suggest that the radiosensitization effects of pIGF-1R inhibitor, linsitinib, were primarily due to the inhibition of DNA repair or augmentation of damage by cycle arrest and induction of apoptosis.

### 3.5. Inhibition of pIGF-1R Reverses Radioresistance in CNE-2G Cells

**Figure 5:**
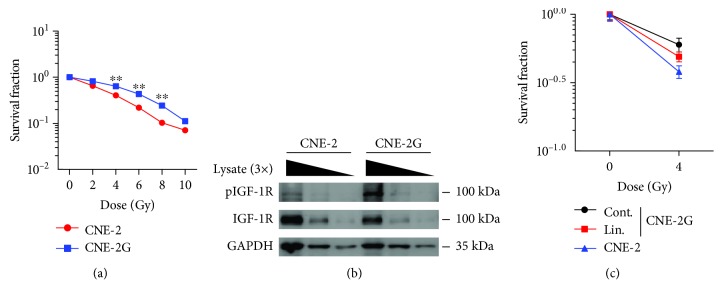
IGF-1R inhibition by linsitinib reverses the radioresistance in NPC cells. (a) Survival curves of parental CNE-2 cell lines and radioresistant CNE-2G cell lines. CNE-2 or CNE-2G cells were seeded in 6-well plates and further irradiated with various doses (0-10 Gy). The cells were cultured for 14 days to allow colony formation. (b) Verification of pIGF-1R expression levels by Western blotting. Serial dilution was used for cell lysate. (c) The clonogenic survival assay of CNE-2 and radioresistant CNE-2G cells; cells were treated with or without linsitinib (Lin., 0.4 *μ*M) and then irradiated with 4 Gy. Survival fraction was determined in the same way as in (a). Each data point represents the mean of three experiments ± SD.

To further provide evidence of targeting IGF/IGF-1R reversing radioresistance in NPCs, we established IR resistant CNE-2 cell line (CNE-2G) by long-term radiation. Colony formation assay was carried out to investigate the survival curves of the parental CNE-2 cell line and the radioresistant CNE-2G cell line. The radiation dose-response of different cells is shown in Figure 5(a), indicating that the CNE-2G survival curves are higher than parental CNE-2. We next compared the phosphorylation of IGF-1R in CNE-2G and its parental cell line. Lysate serial dilution was performed and subjected to Western blotting. Rather than parental cell line, pIGF-1R were increased in CNE-2G cells; however, basal IGF-1R did not change much (Figure 5(b)). Notably, linsitinib treatment partially reversed CNE-2G radioresistance (Figure 5(c)). These results suggest that pIGF-1R takes part in NPC-radioresistant processes and plays a vital role in NPC-radioresistant mechanisms.

## 4. Discussion

Inhibition or depletion of IGF-1R enhances the sensitivity of human cancer cells to IR and cytotoxic drugs in melanoma, prostate, and lung cancer [17, 27, 28]. The variety of cellular responses mediated by IGF-1R is associated with the downstream signaling pathways, including the MAPK and PI3K pathways. In this study, our results showed that the IGF-1R inhibitor, linsitinib, could suppress the ERK and Akt activity and thereby inhibit NPC cell proliferation via inducing apoptosis and cell cycle arrest. Furthermore, we demonstrated that linsitinib could enhance the radiosensitivity of NPC in vitro.

The antitumor activity of linsitinib demonstrated in preclinical models has been evaluated in 86 patients with advanced, treatment-refractory solid tumors [29]. Recently, completed phase II trials have evaluated linsitinib combination therapies with paclitaxel in patients with recurrent ovarian cancer and with erlotinib in metastatic EGFR-mutant non-small-cell lung cancer (NSCLC) patients [30, 31]. Clinical benefit was not observed in linsitinib-treated group, highlighting the urgent need for robust biomarkers in IGFR-targeted therapies. The combination of IGF-1R antibody and radiation has been a challenge in lung cancer and head and neck cancer [32, 33]. The results revealed that IGF-1R antibody increased the sensitivity of NSCLC to radiation in vitro by increasing apoptosis and inhibiting the repair of radiation-induced DNA DSBs. Consistent with these results, our study proved that linsitinib alone slightly induced apoptosis. However, the combination of linsitinib and IR exhibited potent proapoptotic properties. The apoptosis may be caused by persistent DNA damage with IR and IGF-1R inhibitor treatment.

Radiation treatment is effective to prevent the early tumor progression with good prognosis in NPC. However, it does not prevent the development of loco-regional recurrence and distant metastasis, especially when the tumor is in the advanced stage (type III or IV). We and others have shown that upregulation of IGF-1R in human tumor tissues is associated with higher tumor grade and poor survival in gastric cancer, cervical cancer, and NPC [18, 20, 34]. Therefore, aberrantly activated IGF-1R may decrease radiosensitivity in a self-protective feedback loop model in tumor cells. Indeed, IGF/IGF-1R expressions are negatively correlated with 5-year overall survival in several types of cancer [22, 24, 35]. Our data demonstrated that targeting IGF-1R by linsitinib could significantly increase radiosensitivity in both one fractionated cell line and long-term fractionated radioresistant cell line in NPC. The combination of IR and linsitinib remarkably prolonged G2-M cell cycle arrest in NPC cells. This finding is consistent with the previous report that IGF-1R inhibition induces cell cycle arrest in G1 or G2 phase in NSCLC cell lines [36]. Importantly, the combination of IR and linsitinib effectively enhances radiosensitivity in NPC cells. This could be explained by that G2-M phase is the most sensitive cell phase for IR. Although the rationale for the IGF-1R inhibition to increase DNA damage ability has not been well documented, it has been recently reported that depletion of IGF-1R delays repair of radiation-induced DNA DSBs by both nonhomologous end joining and homologous recombination in prostate cell lines [27]. Other studies revealed that ataxia telangiectasia mutated (ATM) or major vault protein (MVP) in cell proliferation and DNA repair pathway probably participate in the modulation of radiation response mediated by IGF-1R [37, 38]. Nonetheless, both pathways probably participate in the modulation of radiation response mediated by IGF-1R.

Radiotherapy is a major treatment modality for NPC. Principles of radiation therapy in primary tumor and involved lymph nodes are that 69.96 Gy (2.12 Gy/fraction) daily from Monday to Friday in 6–7 weeks. Needless to say, local advanced NPC patient could be given higher dose in primary, but normal organs at risk restrict IR dose. To overcome this barrier, intracavitary brachytherapy (ICBT) was used as a boost after IMRT in NPC patient to improve local control [39, 40]. Accordingly, the cumulative dose is 78-82 Gy. To further investigate our hypothesis, we established radioresistant cell line (CNE-2G) by long-term radiation treatment. Using this cell model, we found that linsitinib treatment partially reversed CNE-2G radioresistance. These results suggest that pIGF-1R takes part in NPC radioresistant processes.

Most of the studies have tried to explore the effect of IGF-1R inhibition to elucidate the possible use of IGF-1R as a target in cancer treatment. At the same time, another line of research that leads to evaluate the implications of IGF-1R in radiation resistance was developed, which is trying to develop possible strategies to enhance radiosensitivity. In the line of IGF-1R-mediated radiation resistance, little is known about the mechanisms behind this association. The PI3K/Akt/mTOR pathway is an important intracellular signaling pathway in radioresistance. Increasing evidence showed that dual PI3K/mTOR inhibitors can induce higher radiosensitivities in NPC [41]. Dual PI3K/mTOR inhibitors have a broader efficacy across more genotypes with proapoptotic effects compared with agents targeting only one component in this signaling pathway. Reduction of Raf kinase inhibitory protein (RKIP), in turn increasing downstream ERK expression, has been reported to enhance radioresistance in NPC [42]. High expression levels of pAkt and pERK in human NPC tissue have been shown a correlation with poor radiation response in NPC patient. Thus, upstream protein-targeting agents are theoretically the most potent inhibitors since they lack the downstream feedback activation of MAPK and PI3K. In the present study, we showed that IR alone trends to enhance the phosphorylation of Akt and ERK in NPCs, which was consistent with previous studies. Further inhibiting IGF-1R that is an upstream protein of Akt and ERK sensitized NPCs upon IR by decreasing pAkt and pERK, indicating the usefulness of IGF-1R inhibition for radiosensitivity in NPC.

## 5. Conclusion

Taken together, our results showed that targeting IGF-I/IGF-1R has an antitumor activity and could sensitize radio sensitivity in NPCs. Linsitinib exerted its function on cell proliferation by suppressing IGF-I/IGF-1R axis, consequently inhibiting the downstream phosphorylation of Akt and ERK, delaying cell cycle arrest, and inducing apoptosis. Moreover, these effects further increase radiosensitivity and reverse radioresistance in NPC. These results may provide a new insight into understanding the mechanism of NPC radioresistance, as well as giving a potential therapeutic target for concurrent radiotherapy or TKI in NPC. Further evaluation of IR in combination with linsitinib in clinical trials is warranted to improve the outcomes of patients with NPC.

## Data Availability

The data used to support the findings of this study are available from the corresponding author upon request.
